# Extraction and Integration of Genetic Networks from Short-Profile Omic Data Sets

**DOI:** 10.3390/metabo10110435

**Published:** 2020-10-29

**Authors:** Jacopo Iacovacci, Alina Peluso, Timothy Ebbels, Markus Ralser, Robert C. Glen

**Affiliations:** 1 Department of Metabolism, Digestion, and Reproduction, Faculty of Medicine, Imperial College London, London SW7 2AZ, UK; alina.peluso@gmail.com (A.P.); t.ebbels@imperial.ac.uk (T.E.); 2 The Molecular Biology of Metabolism Laboratory, The Francis Crick Institute, London NW1 1AT, UK; markus.ralser@crick.ac.uk; 3 Charité, Universitätsmedizin Berlin, Charitéplatz 1, 10117 Berlin, Germany; 4 Centre for Molecular Informatics, Department of Chemistry, University of Cambridge, Lensfield Road, Cambridge CB21EW, UK

**Keywords:** similarity measures, mahalanobis cosine, multiplex networks, multi-omics integration, ionomics, metabolomics

## Abstract

Mass spectrometry technologies are widely used in the fields of ionomics and metabolomics to simultaneously profile the intracellular concentrations of, e.g., amino acids or elements in genome-wide mutant libraries. These molecular or sub-molecular features are generally non-Gaussian and their covariance reveals patterns of correlations that reflect the system nature of the cell biochemistry and biology. Here, we introduce two similarity measures, the Mahalanobis cosine and the hybrid Mahalanobis cosine, that enforce information from the empirical covariance matrix of omics data from high-throughput screening and that can be used to quantify similarities between the profiled features of different mutants. We evaluate the performance of these similarity measures in the task of inferring and integrating genetic networks from short-profile ionomics/metabolomics data through an analysis of experimental data sets related to the ionome and the metabolome of the model organism *S. cerevisiae*. The study of the resulting ionome–metabolome *Saccharomyces cerevisiae* multilayer genetic network, which encodes multiple omic-specific levels of correlations between genes, shows that the proposed measures can provide an alternative description of relations between biological processes when compared to the commonly used Pearson’s correlation coefficient and have the potential to guide the construction of novel hypotheses on the function of uncharacterised genes.

## 1. Introduction

The development and reduction in cost of high-throughput technologies in the post-genomic era has made possible genome-wide screening experiments that measure the molecular phenotypes observed in response to single gene alterations, such as deletion, or as a result of an increase in expression of the protein coding sequence [1,2,3,4]. As a consequence, functional omics studies that go beyond the paradigm of functional genomics and that are aimed at investigating genotype–phenotype relations at different omic layers have been carried out. Metabolomic [5,6,7] and ionomic [8,9] profiling have been combined with high-throughput screening of yeast *S. cerevisiae* single-gene deletion libraries to quantify elements and amino acids (essential building blocks of the cell whose concentration is highly informative on the physiological state in response to genetic perturbations), and to subsequently assess correlations between different mutants on the basis of the measured profiles of biological features. Because these molecular or sub-molecular signatures can be mapped and associated to a consistent region of the genome, statistical inference techniques are often applied to extract genetic networks from correlations that will reflect the interplay between gene function, molecular signatures, and environmental factors. Among other methods such as Bayesian networks [10,11] and Gaussian graphical models [12,13,14] reconstruction, relevance network inference [15,16,17,18] is the most used technique, and it is based on the idea of enforcing a pairwise similarity/distance measure between genes in order to extract via similarity-based thresholding a network of genetic associations on which the modern tools and analysis techniques of network theory [19,20,21] can be applied to reveal functional patterns at a system level. To quantify similarities, the Pearson’s correlation coefficient [22,23] is commonly adopted, regardless of the assumptions that the data have to satisfy for it to be used (such as approximate normality and the absence of outliers). This is a practice that is justified by a consensus that its drawbacks are mainly theoretical and that it turns out to be useful in practice in many applications and analyses of real-world data sets [24,25,26,27]. However, comprehensive functional omic approaches that target and simultaneously measure a substantial fraction of the intracellular elements (ionomics), or a complete class of metabolites, (e.g., amino acids) [7], provide a number of features *M* profiled for each mutant that is much smaller than the total number *N* of mutants in the library (*N* order of the genome size), mathematically M<<N. In this paper, we discuss the potential pitfalls of using the Pearson correlation coefficient in this short-profile omics scenario. We propose two extensions of the cosine similarity, namely the *Mahalanobis cosine* similarity and the *hybrid Mahalanobis cosine* similarity that appear more suitable for quantifying phenotype similarities between deletion mutants in the applications described here. Starting from theoretical considerations on the characteristic structure of short-profile omic data, we develop and apply a methodology to quantify advantages these measures may have in the task of extracting biologically meaningful genetic networks. We do this by considering three experimental benchmark data sets of the ionome and the metabolome of the model organism *S. cerevisiae* and several large curated databases as ground truth for genetic annotations. Our testing procedure for the similarity measures evaluates two fundamental aspects of the process of information extraction that accompanies the inference of a genetic network, namely the performance in encoding relevant biological relationships in the resulting network topology, and the ability to capture potential new biological information when integrating networks from different omic layers.

## 2. Materials and Methods

### 2.1. Similarity Measures for Short Omic Profiles

Let *Z* be an N×M matrix describing *N* genome-scale observations of a set of *M* biological signatures (features)—for example, the intracellular concentration of *M* amino acids in *N* different mutant strains of *S.cerevisiae* in which a single open reading frame (ORF) has been deleted, so that N>>M. Let’s assume that these features are standardised, meaning that for each amino acid *j*, the distribution of intracellular concentrations observed across the genome has zero-mean and unit variance:

$$\begin{array}{cc} \mu_{j} & =\frac{\sum_{i=1}^{N}z_{ij}}{N}=0\;\forall j,\\ \sigma_{j} & =\sqrt{\frac{\sum_{i=1}^{N}{\left(z_{ij}-\mu_{j}\right)}^{2}}{N-1}}=1\;\forall j. \end{array}$$

For each molecular signature *f*, we can define a genome-wide feature vector v (columns of the matrix *Z*):

(1) $$v^{f}=\left(z_{1f},\ldots ,z_{Nf}\right),$$

and since each mutant strain is associated to a specific gene *g* in the genome, we can write for each gene its associated profile (rows of *Z*):

(2) $$z^{g}=\left(z_{g1},\ldots ,z_{gM}\right).$$

**Figure A1:**
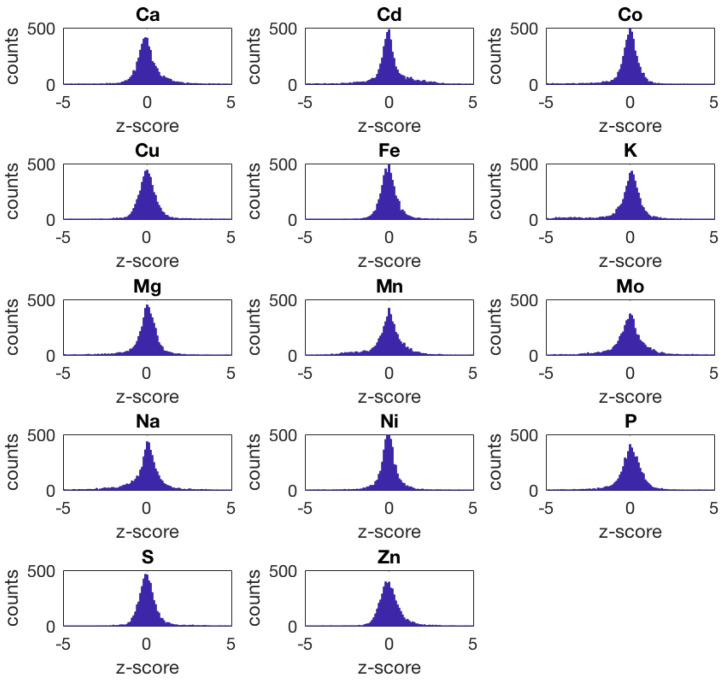
Distribution of the final *z*-scores across all mutants of the Ionome KO data set for each element in the data set. All distributions exhibit long tails. Some distributions appear to be skewed.

**Figure A2:**
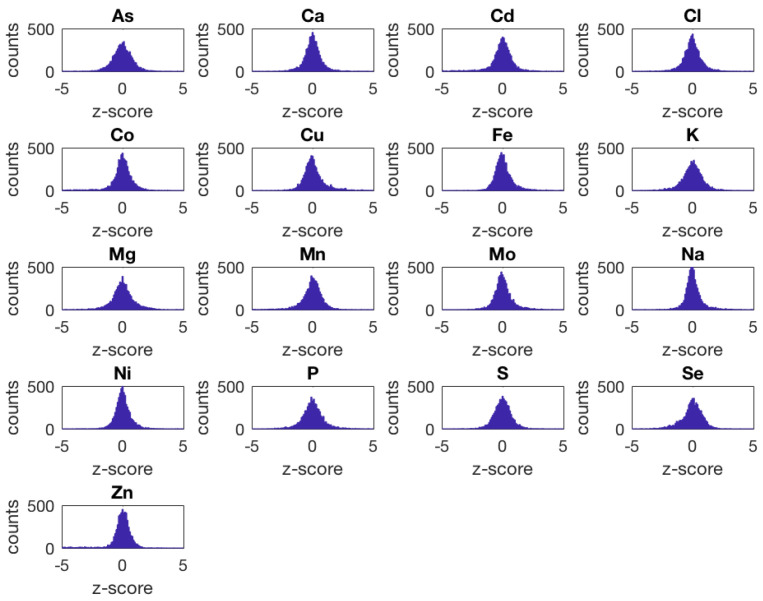
Distribution of the final *z*-scores across all mutants of the Ionome OE data set for each element in the data set. All distributions exhibit long tails. Some distributions appear to be skewed.

**Figure A3:**
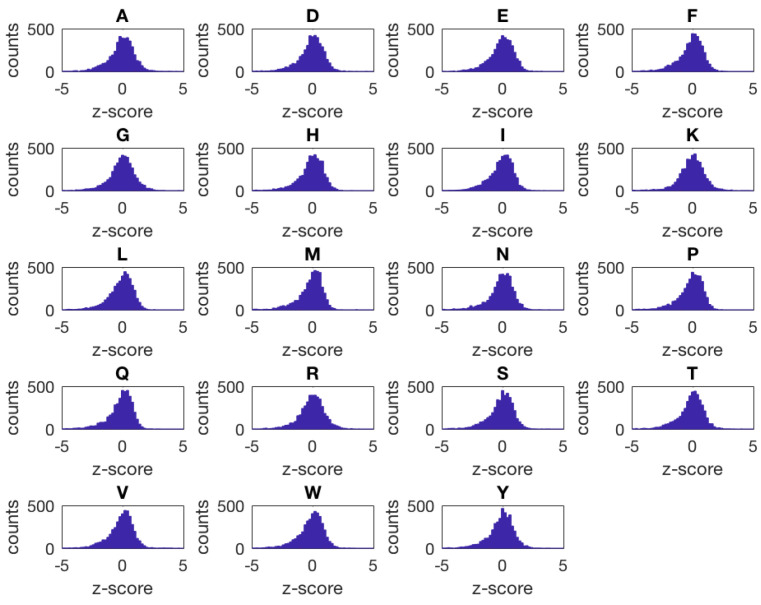
Distribution of the final *z*-scores across all mutants of the Metabolome AA data set for each element in the data set. All distributions exhibit long tails and are highly skewed.

The distribution of the intracellular concentration values of amino acids and other metabolism-related biomolecules measured across a collection of all possible specific mutants (single-gene knockout or overexpression) is usually centred around a typical value corresponding to the wild type phenotype (most of the mutations are neutral and produce a phenotype consistent with the one of the wild type), and exhibits long tails due to high/low concentration values associated to those minority of mutations that alter the cell function and produce a phenotype detected at the level of the molecular profile (examples of these distributions are shown in Figure A1, Figure A2 and Figure A3). Moreover the concentrations of these biomolecules are likely to vary in a correlated fashion because they are regulated by the network of metabolic reactions and functional processes within the cell. Therefore, we assume for our omic data set that the standardised feature vectors $v^{f}$ are skewed (H1) and that some of them can be highly correlated (H2):

(3) $$H1:|\frac{\frac{1}{N}\sum_{i=1}^{N}{\left(v_{i}^{f}-{\bar{v}}^{f}\right)}^{3}}{{\left[\frac{1}{N-1}\sum_{i=1}^{N}{\left(v_{i}^{f}-{\bar{v}}^{f}\right)}^{2}\right]}^{\frac{3}{2}}}|>>0\;\forall f,$$

and

(4) $$H2:corr\left(v^{f},v^{f^{\prime }}\right)=\left|\frac{\left(v^{f}-{\bar{v}}^{f}\right)\left(v^{f^{\prime }}-{\bar{v}}^{f^{\prime }}\right)}{\sqrt{{\left(v^{f}-{\bar{v}}^{f}\right)}^{2}}\sqrt{{\left(v^{f^{\prime }}-{\bar{v}}^{f^{\prime }}\right)}^{2}}}\right|>>0,\;\mathrm{for}\mathrm{some}f,f^{\prime }.$$

**Figure 1:**
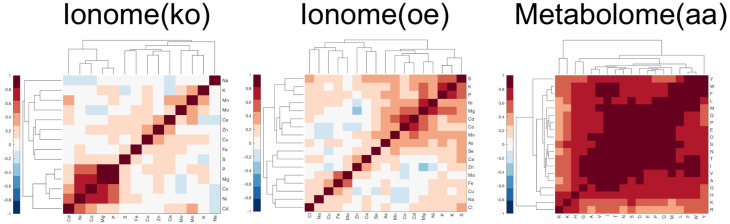
Patterns of feature–feature correlations observed in the feature correlation matrix (Pearson coefficient) for the three experimental benchmark data sets considered in this study. In the ionome data sets, the features correspond to intracellular concentrations of different elements profiled in diverse *S. cerevisiae* mutant strains, while in the metabolome data set, the features correspond to the intracellular concentrations of amino acids.

A way to put hypothesis H2 in practical terms is to say that, when measuring from the data the feature–feature covariance matrix *C*, we expect to find a substantial fraction of the matrix elements to be significantly larger than zero in absolute value; therefore, the features reveal a significant and extended pattern of pairwise correlations (examples of these patterns can be seen is Figure 1).

Taking into account these factors, each element of a generic profile $z^{g}$, corresponding to the concentration value of a specific amino acid, is associated with a distinct, characteristic non-Gaussian, skewed empirical distribution, and the values in the profile array are correlated. It follows that the average value of the profile ${\bar{z}}^{g}$ is likely to be substantially different from zero for a consistent fraction of mutants showing a phenotype (see Appendix C for a systematic analysis of the average profile value on simulated short-profile omic data). Therefore, if our goal is to find a reliable pairwise similarity score between mutant profiles to infer genetic associations from the data, the Pearson correlation coefficient (PCC) [22,23] might not be the best choice. For two genetic profiles $\left(z^{g},z^{g^{\prime }}\right)$ the similarity in terms of PCC is given by:

(5) $$PCC\left(z^{g},z^{g^{\prime }}\right)=\frac{\sum_{j=1}^{M}\left(z_{gj}-{\bar{z}}^{g}\right)\left(z_{g^{\prime }j}-{\bar{z}}^{g^{\prime }}\right)}{\sqrt{\sum_{j=1}^{M}{\left(z_{gj}-{\bar{z}}^{g}\right)}^{2}}\sqrt{\sum_{j=1}^{M}{\left(z_{g^{\prime }j}-{\bar{z}}^{g^{\prime }}\right)}^{2}}}.$$

PCC is intrinsically multivariate (because of the arithmetic mean ${\bar{z}}^{g}$), and it should be applied under the assumption that the vectors $z^{g}$ contains uncorrelated values that are consistent with the same marginal Gaussian distribution. When we drop the multivariate terms, we have the cosine similarity, defined as:

(6) $$cos\left(z^{g},z^{g^{\prime }}\right)=\frac{\sum_{j=1}^{M}z_{gj}z_{g^{\prime }j}}{\sqrt{\sum_{j=1}^{M}z_{gj}^{2}}\sqrt{\sum_{j=1}^{M}z_{g^{\prime }j}^{2}}}.$$

**Figure A4:**
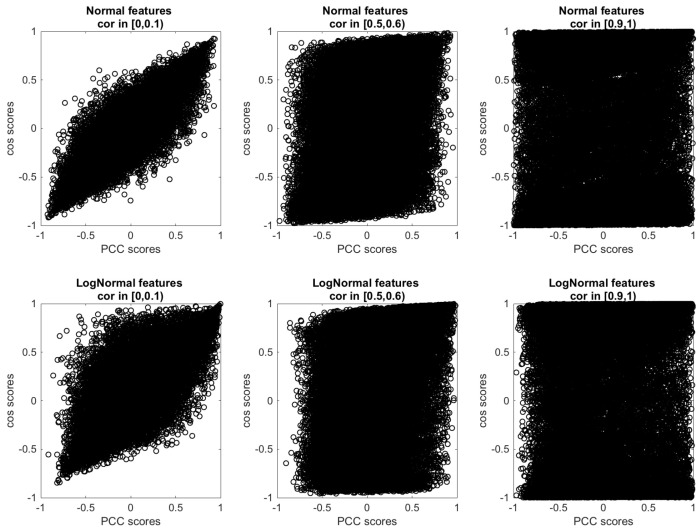
Scatter plots of the profile similarity scores measured via PCC against those measured via cos on synthetic data sets of n=300 profiles of M=10 features for different level of features’ correlation. Top row: features follow a Normal distribution. Bottom row: features are distributed according to a log-Normal distribution (*skewness* ≥1.5).

**Figure A6:**
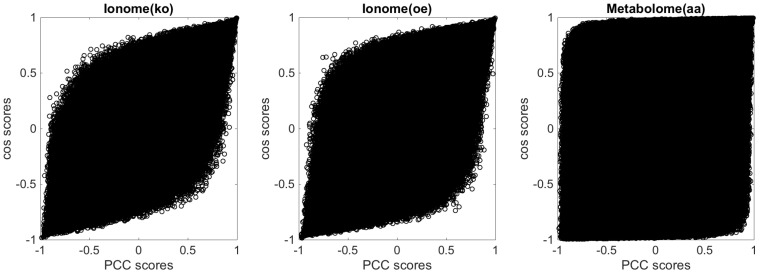
Scatter plots of the profile similarity scores obtained with PCC against those obtained with cos for the three experimental data sets used as benchmarks in this study.

If we see z as a generic vector in an *M*-dimensional space, then $cos(z^{g},z^{g^{\prime }})$ represents the angle between the two vectors $z^{g}$ and $z^{g^{\prime }}$. If our features *f* were Gaussian-like, standardised, and uncorrelated, then the values of the gene profiles would also be normally distributed, and in that scenario, for most of the genes, we would have ${\bar{z}}^{g}≃0$ and $PCC\left(z^{g},z^{g^{\prime }}\right)≃cos\left(z^{g},z^{g^{\prime }}\right)$; therefore, there would be no reason to prefer the cosine over the PCC and vice versa (see Figure A4 and Figure A6 for a comparison between cos- and PCC-generated similarity scores from real short-profile omic data sets and from synthetic short-profile omic data).

The cosine similarity formula contains the Euclidean norm in the denominator; indeed, all Euclidean vector spaces are inner product spaces in which the notion of *angle* between two generic vectors is well defined (for more details, see Appendix A). Now we consider the following metric, the Mahalanobis distance [28], defined as:

(7) $$d\left(z^{g},z^{g^{\prime }}\right)=\sqrt{\left({\left(z^{g}\right)}^{T}C^{-1}z^{g^{\prime }}\right)}$$

where *C* is the feature–feature covariance matrix estimated from the data. Since any covariance matrix is always semi-positive definite, it is easy to show (see Appendix A) that the Mahalanobis metric induces an inner product space and that it is perfectly legitimate to write the following Mahalanobis cosine similarity:

(8) $$cosM\left(z^{g},z^{g^{\prime }}\right)=\frac{\sum_{j=1}^{M}z_{gj}^{\prime }z_{g^{\prime }j}^{\prime }\lambda_{j}^{-1}}{\sqrt{\sum_{j=1}^{M}{z_{gj}^{\prime }}^{2}\lambda_{j}^{-1}}\sqrt{\sum_{j=1}^{M}{z_{g^{\prime }j}^{\prime }}^{2}\lambda_{j}^{-1}}}$$

where $z_{gj}^{\prime }$ are the elements of the profile vector $z^{g}$ in the base where the covariance matrix is diagonal with eigenvalues $\lambda_{j}$. We found one application of the Mahalanobis cosine in computer vision (face recognition) [29] and, to our knowledge, this measure has never been applied in the field of bioinformatics or computational biology before. In this paper, we show that it is a suitable and powerful measure to extract genetic association networks from short-profile omic data sets. As an analogue to the *pseudo-cosine* [30], we also introduce the hybrid Mahalanobis cosine similarity, defined as:

(9) $$cosH\left(z^{g},z^{g^{\prime }}\right)=\frac{\sum_{j=1}^{M}z_{gj}^{\prime }z_{g^{\prime }j}^{\prime }}{\sqrt{\sum_{j=1}^{M}{z_{gj}^{\prime }}^{2}\lambda_{j}^{-1}}\sqrt{\sum_{j=1}^{M}{z_{g^{\prime }j}^{\prime }}^{2}\lambda_{j}^{-1}}}.$$

The cosH is not a proper cosine function because the inner product in the formula corresponds to the Euclidean dot product while the norms are computed with the Mahalanobis norm. Therefore, in principle, cosH is not bounded between −1 and 1. However, for practical applications, it is always possible to rebound the scores in the range [−1, 1] by dividing all the scores extracted from a given data set by the largest absolute score value. Equations (8) and (9) show how the two proposed similarity measures enforce information from the empirical feature–feature covariance matrix extracted from the entire set of data (in the N>>M framework, the estimation of this matrix is accurate). When computing each pairwise similarity score between profiles, these measures take into account the geometry of the cloud of data points in the *M*-dimensional feature space (the covariance eigenvalues can be seen to describe a hyper-ellipse with axes of lengths $\left\{\lambda_{i}\right\}$), and, in analogy with the general relativity formalism [28], they dilate distances by the factors $\lambda_{j}^{-1}$ so as to penalise the directions in the feature space along which the covariance is low and the data points are less scattered.

### 2.2. Omic Benchmark Data Sets

Throughout the paper, we evaluate and compare the performance of PCC, cos, cosM, and cosH in inferring genetic relevance networks from short-profile omic data sets. In particular, we focus on testing these measures in two fundamental tasks of omics data analysis, namely the retrieval of known biological information, and the detection of potential undiscovered functional associations between genes. To do that, we consider here three experimental benchmark data sets:

(1) Yeast ionome ko (non-essential ORF knock-out mutants, iHUB [31,32,33]).
  The yeast ionome knock-out (ko) data set contains population-average intracellular concentrations of 14 different elements (Ca, Cd, Co, Cu, Fe, K, Mg, Mn, Mo, Na, Ni, P, S, and Zn) quantified by means of inductively coupled plasma–mass spectrometry (ICP–MS) for a library of 4944 *S. cerevisiae* haploid mutant strains having a single non-essential open reading frame knocked out.This data set includes a total of 26,976 samples measured in 305 different plates. Most of the strains were measured in replicates of four (4207), 684 strains in replicates of eight, 48 strains in replicates of 12, and two strains in replicates of 16. Additionally, three control strains present in multiple trays were included in our analysis: YDL227C, 1620 replicates; YLR396C, 1224 replicates; YPR065W, 1224 replicates.
(2) Yeast ionome oe (overexpression mutants, iHUB [31,32,33]).
  The yeast ionome overexpression (oe) data set contains population-average intracellular concentrations of 17 different elements (As, Ca, Cd, Cl, Co, Cu, Fe, K, Mg, Mn, Mo, Na, Ni, P, S, Se, and Zn) quantified by means of inductively coupled plasma–mass spectrometry (ICP–MS) for a library of 5718 *S. cerevisiae* haploid mutant strains having a single essential or non-essential open reading frame overexpressed. This data set includes a total of 24,060 samples measured in 310 different plates. Most of the strains were measured in replicates of four (5426), 287 strains in replicates of eight, and five strains in replicates of 12.
(3) Yeast metabolome aa (amino acid profile of non-essential ORF knock-out mutants [7]).
  The yeast metabolome (aa) data set contains population-average intracellular concentrations of 19 different amino acids (A, D, E, F, G, H, I, K, L, M, N, P, Q, R, S, T, V, W, Y) quantified by means of liquid chromatography–mass spectrometry (LC–MS) for a library of 4475 *S. cerevisiae* haploid mutant strains having a single non-essential open reading frame knocked out. This data set includes a total of 4653 samples measured in 12 different batches. Most of the strains are associated with a single sample (4324), 128 strains have two replicates, 19 strains have three replicates, and four strains have four replicates.

Information and details concerning these data sets, including quality control analyses, can be found in [7,33,34]. To allow for an unbiased comparison of the different measures performance across the different data sets, all data are processed using the same pipeline starting from the raw measured concentration values. The pipeline corrects the raw data for batch effects and extracts a characteristic concentration profile for each strain by sequentially applying the following operations: (i) log-transformation of the data, (ii) median plate normalisation, (iii) outlier detection and removal, (iv) extraction of a median profile from the replicates (where replicates are available) for each strain, and (v) standardisation of the concentration values (a detailed description of the pipeline can be found in Appendix B). For each data set, the final mutant-related feature profiles show how many standard deviations the concentrations deviate from the median concentration measured across all the strains in that data set. These data sets have been obtained using a similar experimental design, and they also present the general characteristic of short-profile omic data sets discussed in Section 2.1: in Table 1, we report the average absolute feature skewness (AAFS) and the number-of-features over number-of-genes ratio M/N (after data processing); in Figure 1, we show the patterns of feature–feature correlations extracted from each experimental data set using the Pearson correlation coefficient (note that in the the framework N>>M, the feature–feature covariance matrix can be reliably estimated from the empirical correlation matrix).

### 2.3. Genetic Networks Inference

The statistic of pairwise similarity score values depends in general on the specific similarity measure used to calculate the scores. In order to robustly compare the topology of the genetic relevance networks obtained with the different similarity measures of interest (PCC, cos, cosM, cosH) we adopt a rank-based thresholding of the scores that depends in turn on the more robust rank statistic of the scores. Our general inference methodology consists of the following steps:

- Score computation: we compute all pairwise similarity scores between the *N* genes of a given data set with each similarity measure of interest.
- Score ranking: we rank in descending value order each set of scores computed with a different similarity measure.
- Relevance network extraction: we retain the top-*n* ranked scores in each set to define for each similarity measure a genetic association network of *N* nodes and *n* links; the links correspond to the *n* highest values in the score rank statistic.

**Figure 2:**
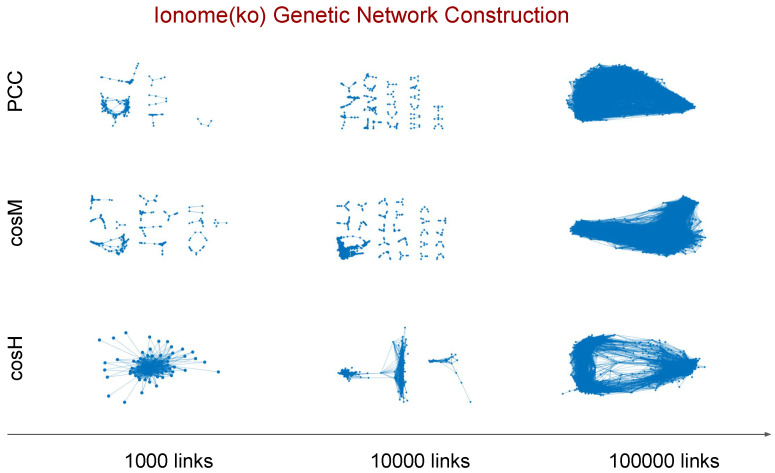
Genetic relevance networks extracted from the ionome knock-out data set using three different values of the relevance threshold *n*. For a given threshold value, *n* links included in the network correspond to the top *n* pairwise similarity scores measured between the genes. To improve visualisation, we display the connected subgraphs with a size larger than or equal to five nodes when PCC, cosM, and cosH are used to measure the scores, respectively. The evolution of the network topology resulting from the inclusion of more and more links for decreasing *n* values can be regarded as a reverse percolation process on the network.

This procedure allows for a robust comparative analysis of the genetic networks inferred using the different measures at any fixed value of the relevance threshold *n*. In Figure 2, we show the topology of the genetic relevance networks inferred from the ionome (ko) data set for different values of the relevance threshold *n* when using PCC, cosM, and cosH. To improve visualisation, we plot only the connected subgraphs with a size larger than or equal to five nodes. An analysis of the false positive rate of associations for the inferred relevance networks is reported in Appendix D.

### 2.4. Multiplex Integration of Genetic Networks

Once we have defined the procedure to extract genetic networks from a single omic data set, we can integrate, within the multilayer network paradigm, the genetic associations from all three benchmark data sets. In particular, our integration methodology will focus on constructing multiplex networks [35,36,37,38]. For each of the similarity measures of interest, we will take the set of all genes profiled in at least one data set and we will construct a multiplex network of three layers, each containing the single network inferred from one of the data sets by retaining the top 100,000 most relevant associations.

**Figure 3:**
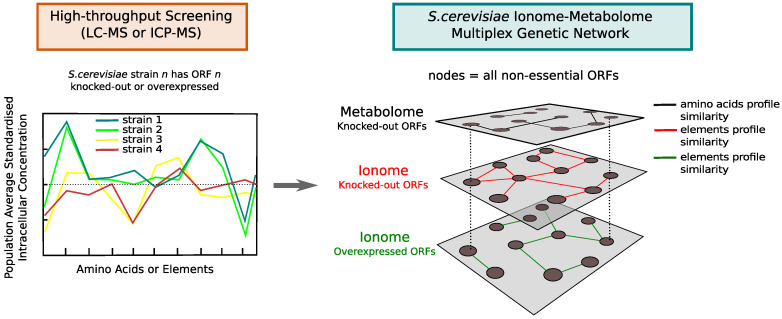
Diagram illustrating the concept of the *S. cerevisiae* ionome–metabolome multiplex genetic network. By means of mass-spectrometry technology, it is possible to profile the intracellular concentration of amino acids and elements in yeast mutant strains that have one single open reading frame (ORF) deleted or overexpressed (loss or amplification of single gene function). By quantifying similarities between mutant profiles, a genetic network can be extracted from each high-throughput screening and the single omic networks can be integrated into a multilayer network where the same set of nodes (corresponding to almost all non-essential ORFs) is connected on different layers, each describing connections between genes whose loss/overexpression produce a similar phenotypic response at the level of the metabolome or ionome.

A multiplex network G is mathematically defined by a set of nodes *V* and *K* single-network layers $G^{\alpha }=\left(V,E^{\alpha }\right)$, with α=1,2,…,K and $E_{\alpha }$ indicating the set of links in layer α (see Figure 3). The multiplex network framework allows us to efficiently encode associations between genes for which there is omic-specific evidence or evidence across multiple omic layers by means of the *multilink* concept [38,39,40]. A multilink that connects a pair of nodes (i,j) is defined by a vector ${\vec{m}}^{ij}=\left(m_{1}^{ij},m_{2}^{ij},\ldots ,m_{K}^{ij}\right)$ which specifies all the layers α where those nodes are connected ($m_{\alpha }^{ij}=1$), and where they are not ($m_{\alpha }^{ij}=0$). The multilinks can characterise all the different ways in which a pair of nodes in the multiplex can be connected across the *K* layers. For example ${\vec{m}}^{ij}=\left(1,0,0\right)$ indicates that node *i* and *j* are connected in layer 1 only, while in the case ${\vec{m}}^{ij}=\left(1,0,1\right)$, they are connected in both layer 1 and 3. It is then possible to define the *multidegree* $k_{i}^{\vec{m}}$ of a generic node *i* as the number of neighbours that are connected via multilinks of type $\vec{m}$.

## 3. Results

**Figure 4:**
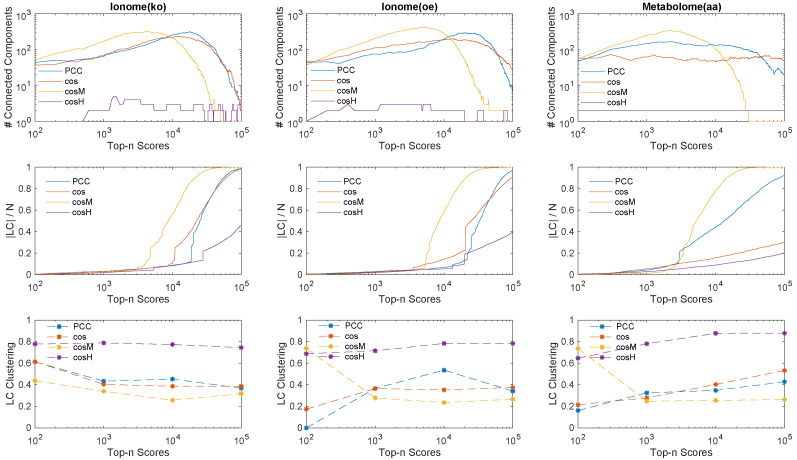
Percolation analysis of the relevance network inference process carried out with all the similarity measures considered, on all experimental data sets under study. (**Top**) we plot for each data set the evolution of the number of connected components in the extracted networks in function of the number of links *n* inferred using the top *n* similarity scores. (**Middle**) We also show the evolution of the size of the largest component (LC) divided by the total number of genes *N* in function of the number of top *n* scores. (**Bottom**) We quantify the average local clustering coefficient of the nodes in the largest component (LC) when different orders of magnitude of number of links are inferred in the genetic network. The curves indicate that cosH tends to connect faster than the other measures all the genes in the network in one connected component from many small subgraphs, while, on the contrary, cosM tends to infer links which characterise relations between genes within few large connected components which are comparable in node size with respect to the largest component and reveal a highly clustered architecture in terms of associations.

The process of inference of a genetic network from a given data set while gradually increasing the relevance threshold (Figure 2) can be regarded as a reverse percolation process on the network, in which edges are sequentially added one at a time between the same set of *N* vertices [41,42]. This fact allows us to obtain insights about the inference power and performance of the similarity measures based on network theory considerations. Our first analysis focuses on three network descriptors, namely (i) the number of connected components—that is, the number of connected subgraphs in the network; (ii) the size of the largest component (LC)—that is, the number of nodes present in the largest connected component of the graph; and (iii) the average local clustering coefficient [43,44] of the largest component—that is, the average fraction of triangles over triplets closed by a node in the LC. The clustering coefficient measures how well the nodes in a graph tend to cluster together, and it works as an indicator for the network modularity [45,46,47]. In Figure 4, we plot the number of connected components, the size of the LC, and the average clustering coefficient of the LC measured from the inferred networks in the top *n* genetic associations ranked according to the different measures considered. Interestingly, the curves reveal that:

- By increasing the relevance threshold *n* and considering more and more links in the inferred networks, cosM tends to generate faster than PCC and than cos a giant component whose node size is a finite fraction of the genome size *N* (yellow curves in top and middle panels, the number of connected components goes to one, and the size of the LC increases asymptotically to *N*), with a relatively (comparable to PCC and cos) low level of clustering of the nodes (bottom panels).
- On the other hand, cosH tends to aggregate genes in few connected components or modules (purple curves, top panels) which grow in parallel with relatively comparable sizes (middle panels, the size of the LC is far below *N*), and are highly clustered (purple marks in bottom panels, high clustering coefficient).

This analysis suggests that the measures that enforce information from the feature covariance matrix, namely cosM and cosH, represent two alternative ways of operating at the genome scale: given a certain number *n* of reliable associations, cosM is able to infer relations that involve a larger set of genes, thus retrieving faster biological information at the global genetic network scale, while cosH can best retrieve information at the local genetic network scale by selecting associations within several isolated clusters of genes.

### 3.1. Retrieval of Known Biological Information

The above considerations are based on the assumption that the links inferred contain relevant biological information. We therefore designed a testing regime that quantifies the performance of the proposed measures in the task of retrieving previously known biological information. By assuming that a certain amount of known biological information is contained in each of the benchmark data sets, we want to quantify which similarity measure can better recall that information. We consider separately five curated databases as ground-truth for genetic associations:

(1) Protein–Protein Interactions (PPI) from STRING [48] (v11, database scores only).
(2) BIOGRID [49] (v3.4.158, genetic interactions only).
(3) Protein Complex Consensus [50] (co-occurrence in protein complexes).
(4) KEGG Biological Pathways [51] (Release 90.1, co-occurrence in metabolic pathways).
(5) Yeast Net Gold Standard [52] (v3, Gene Ontology based gold-standard associations).

For each of the benchmark data sets, we compute the percentage of corresponding ground-truth links that are found in the associated networks (true positive rate, or *recall*) extracted at different values of the relevance threshold *n*, and compare those numbers across the similarity measures of interest used in the inference process. Of note, this approach is conceptually different from a receiver operating characteristic curve analysis (a study of the true positive associations rate against false negative associations rate in function of the threshold *n*), that would evaluate the diagnostic ability of each omic data set in recovering each specific type of biological associations alone. For any set of ground-truth associations (single database), such analysis would disregard any amount of potential new information extracted from a given data set (which is highly dependent on the specific type of omic data considered and on the associated experimental conditions—for example, the type of media used to grow the yeast mutants) as well as information that is relative to other databases.

**Figure 5:**
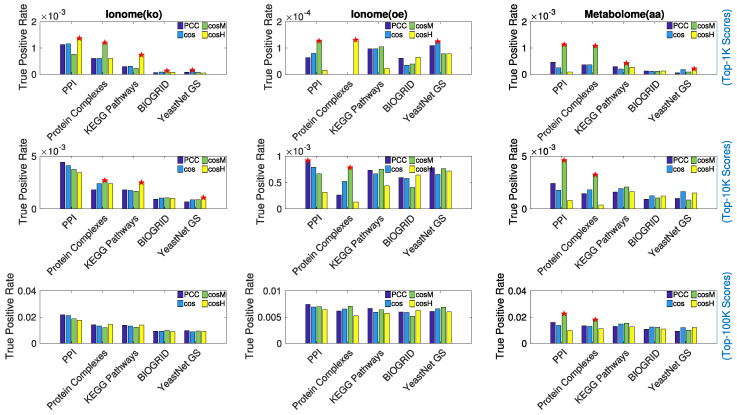
Comparison in performance of the similarity measures under study in recalling previously known biology. Protein–protein interactions (PPI), co-occurrence in protein complexes, co-occurrence in metabolic pathways (KEGG), genetic interactions (BIOGRID), and associations based on Gene Ontology (GO) Terms (YeastNet GS) were considered separately as ground-truth for genetic associations. For each experimental data set of ionome/metabolome, the true positive rate of associations found in the top 1000 scores (**Top**), in the top 10,000 scores (**Middle**), and in the top 100,000 scores (**Bottom**) is reported, respectively, for each of the similarity measures under study. The red stars mark the cases in which the highest true positive rate differs by at least 10% with respect to the second highest. We considered this difference in percentage as the gain in performance associated to the best performing measure. cosM appears to consistently outperform the other measures in recalling protein–protein interactions, protein complexes associations, and biological pathways co-occurrence in the networks extracted from the metabolome (aa) data set.

Results are shown in Figure 5. For the top *n* scores values considered, n∈[1000,10,000,100,000], the true positive rate (TPR) of retrieved associations is reported. The red stars mark the measures that performed best with at least a 10% gain in performance with respect to the second best (that is, the difference in TPR value between the best measure and second best measure divided by the second best TPR). In Table 2, we report details of the best recall performance with its associated performance gain. Overall, the true positive rates are very low, which is expected because it is hard to reconstruct the exact ground truth networks, especially using 1000 or 10,000 top scores; however, the estimated false positive rates of associations are at least an order of magnitude smaller (see Appendix D). In the case of the metabolome (aa), which revealed the most extended and prominent correlation pattern across the set of features (Figure 1), cosM appeared to consistently outperform the other metrics in recalling protein–protein interactions, protein complexes associations, and biological pathways co-occurrences (links between genes annotated to the same metabolic pathway). In the case of the ionome data sets, in which the level of correlation between features was found to be lower, we observed that for the top 100,000 associations considered, the recalling of the similarity measures is comparable; however, when considering lower relevance thresholds (1000 and 10,000 associations), cosM and cosH outperformed the other measures in several cases. For the ionome (ko), cosM and cosH consistently performed best in recalling information about protein complexes and biological pathways, respectively. In particular, when only a few highly reliable associations were considered (top 1000 scores), the gain in performance of cosH and cosM was often extremely high (see Table 2). The Pearson correlation coefficient appeared to perform effectively better only in one single case (PPI, top 10,000 scores).

### 3.2. The Ionome–Metabolome Multiplex Genetic Network of the Yeast *S. cerevisiae*

**Figure 6:**
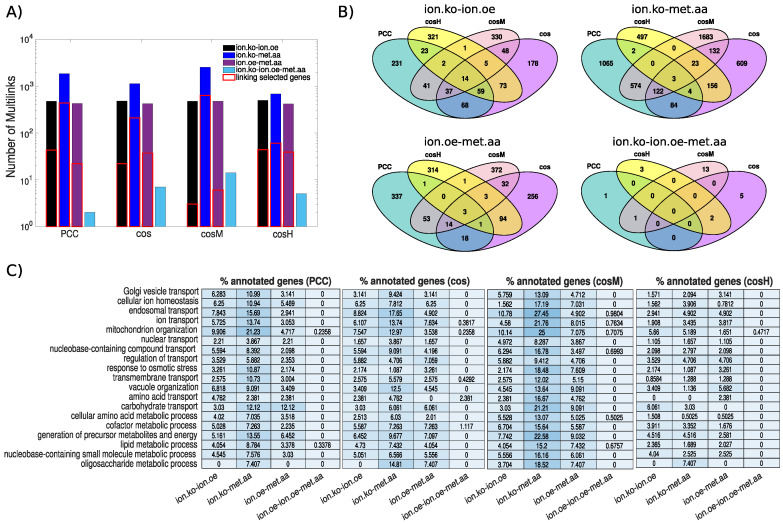
Analysis of the *S. cerevisiae ionome–metabolome multiplex genetic network*. Using each of the similarity measures under study, we construct a multiplex network by superimposing the three networks defined by the top 100,000 similarity scores in the ionome (ko), ionome (oe), and metabolome (aa), respectively. (Panel **A**) The statistics of non-layer-specific multilinks compared across the multiplex networks obtained with the different measures under study. (Panel **B**) Venn diagrams showing the overlap of non-layer-specific multilinks between the different measures under study. (Panel **C**) We show the percentage of genes annotated to a selected subset of GOSlim biological processes related to metabolic processes and cellular ion homeostasis maintenance that are connected in the non-layer-specific multilinks subnetwork of the different measures. In panel A, the number of multilinks of different types that are incident in these selected genes is also highlighted (red solid lines).

When we constructed the multiplex network from the three single-network layers inferred from each data set using the top 100,000 significant associations, we observed that the multilink statistics did not differ in terms of orders of magnitude across the similarity measures considered (Figure 6A), with the exception of the multilinks of the type *ionome.oe-metabolome.aa* for which cosM provided the largest number of links (2537) and cosH the lowest (685). Each measure, however, had a substantial fraction of characteristic multilinks that did not overlap with the other measures (Figure 6B). The lowest specificity was observed for PCC with only 26.8% of characteristic *ionome.ko-metabolome.aa* multilinks, while the highest specificity was observed with cosM (respectively 92.9% of *ionome.ko-ionome.oe-metabolome.aa* multilinks and 77.8% of *ionome.oe-metabolome.aa* multilinks that are not in common with other measures). Furthermore, we checked the content of relevant biological information of non-layer-specific (NLS) multilinks by considering a subset of GOSlim Biological Process Ontology terms from the SGD database [53] that are related to metabolic processes and cellular ion homeostasis maintenance [34,54]. When we looked at the four subnetworks defined by each specific class of NLS multilinks, we found that those constructed with cosM included the largest number of genes annotated to the ontology categories considered (Figure 6C). cosM also yielded the largest number of *ionome.ko-metabolome.aa* multilinks between the annotated genes considered, while for the two multilinks classes *ionome.oe-metabolome.aa* and *ionome.ko-ionome.oe*, cosH revealed the largest number of connections among the annotated genes (Figure 6A).

**Figure 7:**
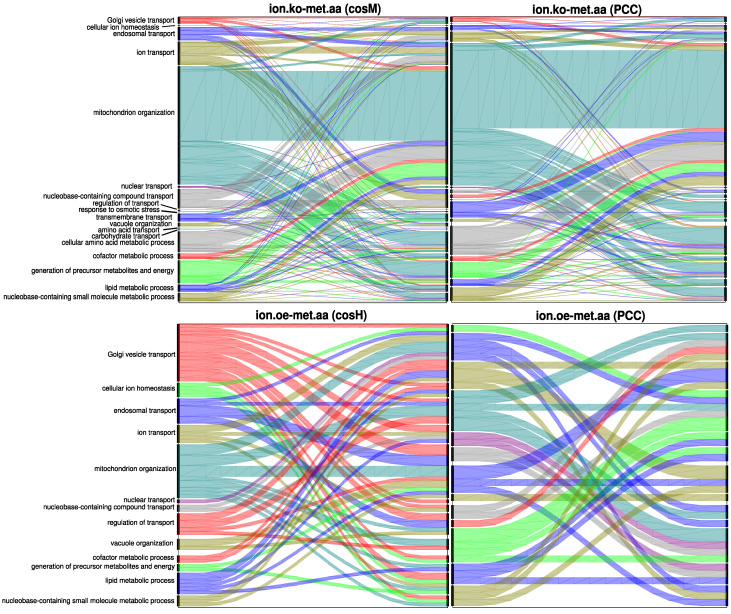
Flow diagrams representing the proportion of multilinks between genes annotated to selected GOSlim classes of biological processes related to ion transport and metabolism. The proposed similarity measures are able to reveal new levels of functional associations between genes: (**Top**) The flows generated by cosM and by PCC for the *ionome.ko-metabolome.aa* multilinks are compared. (**Bottom**) The flows generated by cosH and by PCC for the *ionome.oe-metabolome.aa* multilinks are compared. Those are the classes of multilinks for which cosM and cosH respectively produce the highest flow diversity with respect to PCC.

These results obtained in the multiplex framework were consistent with the previous results and supported the observation that cosM best captures global information at the genome scale by spanning in its NLS subnetworks the largest number of annotated genes, while cosH could reveal larger, well-clustered neighbourhoods of fewer annotated genes, thus better characterising them at a local network scale. The proposed measures also revealed alternative relations between different biological processes compared to the Pearson’s correlation coefficient. In Figure 7, the flow of multilinks between the different ontology classes selected for the *ionome.oe-metabolome.aa* cosH subnetwork and for the *ionome.ko-metabolome.aa*cosM subnetwork is depicted, together with the analogous PCC flows. The large number of genetic associations belonging to these two NLS multilink types (see Figure 6A) also reflected a richer scenario in terms of the flow of multilinks between biological processes. cosM revealed, for example, associations between ion transport genes and genes annotated to endosomal transport, nucleobase-containing compound transport, and vacuole organization, while cosH revealed associations between Golgi vesicle transport genes and several ontologies including Golgi vesicle transport, endosomal transport, ion transport, mitochondrion organization, nucleobase-containing compound transport, transmembrane transport, the cellular amino acid metabolic process, generation of precursor metabolites and energy, and the lipid metabolic process that were not captured by the PCC.

**Figure 8:**
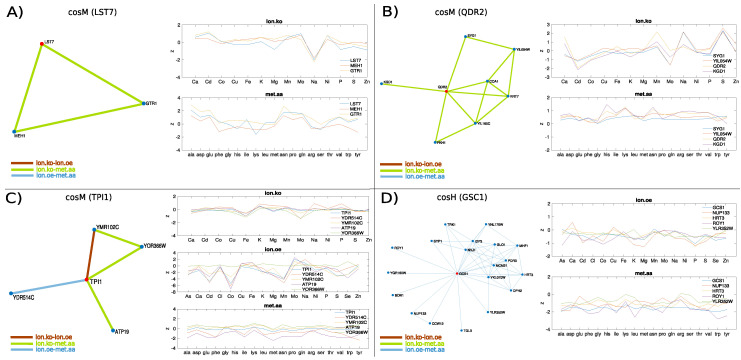
Examples of genes used for hypothesis construction via multiplex neighbourhood analysis. Only non-layer-specific multilinks are plotted, as they represent evidence detected across multiple omic layers (respectively, *ionome.ko-ionome.oe*, *ionome.ko-metabolome.aa*, and *ionome.oe-metabolome.aa*). The non-layer-specific multilink neighbourhood of genes LST7 in the cosM multiplex (Panel **A**), QDR2 in the cosM multiplex (Panel **B**), TPI1 in the cosM multiplex (Panel **C**), and GCS1 in the cosH multiplex (Panel **D**) are shown together with their ion/amino acid profiles.

Figure 8A provides a clear example of a gene, LST7, whose functional role falls at the interface between metabolic processes and ion homeostasis, that is highlighted by the multilink flow analysis. The *ionome.ko-metabolome.aa* neighbourhood of LST7 spanned by cosM (Figure 8A, left) contains GTR1 and MHE1 (EGO1). This triad is known to be involved in the regulation of TOR signalling; indeed, Gtr1 and its membrane-tethering subunits Ego1 are part of the EGO complex that binds the vacuolar membrane and subunits of TORC1. The Lst4-Lst7 complex has been recently shown [55] to attach to the vacuolar membrane next to the GTPase complex Gtr1-Gtr2 under amino acid starvation, and to transiently interact with Gtr1-Gtr2, thereby entailing TORC1 activation and Lst4-Lst7 release from the membrane, under subsequent amino acids stimulation (for example, glutamine). This machinery is reflected by the significantly altered amino acid profiles of the genes (Figure 8A, bottom-right) with an overabundance of several species, including glutamine, and none of the ions overrepresented (Figure 8A, top-right), suggesting that the loss of function of these genes induces in the cell a state of stationary amino acids stimulation. The ion profiles also shows a significant decrease in sodium concentration in the cell that reflects an interplay between the amino acid induced signalling of TORC1 and pH homeostasis: physical interactions from high-throughput data of the protein-fragment complementation assay [56] indeed suggest association of Ego1 and Gtr1 with Vma8, the D subunit of the V1 peripheral membrane domain of the vacuolar ATPase (V-ATPase) that is essential for maintaining ion homeostasis. In humans, Gtr1 signalling has also been shown to depend on interactions with the vacuolar V-ATPase [57,58]).

Finally, in order to illustrate how enforcing these measures can help construct novel hypotheses on the function of uncharacterised genes, we selected examples of genes from the characteristic flows discussed above that contained in their network neighbourhood genes of unknown function (Figure 8B–D). The cosM *ionome.ko-metabolome.aa* neighbourhood of the plasma membrane transport gene QDR2 (Figure 8B, left) suggests an association with the plasma membrane gene SYG1 of unknown function which is also connected with the uncharacterised membrane protein YIL054W. Interestingly, the knockout ionome profiles of QDR2 (which is known to contribute to potassium homeostasis and whose expression is regulated by copper), SYG1, and YIL054W display evident signatures including a characteristic decrease in cadmium and an increase in sulphur, and variable levels of accumulation of manganese and sodium and decumulation of molybdenum. The metabolic profiles show a general overabundance of all amino acid species including leucine and lysine. When we performed transcription factor enrichment analysis (YeastEnricher online tool [59]), we consistently found significant regulation by CAD1, a cadmium resistance gene, involved in stress responses and iron metabolism (adjusted *p*-value 0.04956) and LEU3, involved in amino acids biosynthesis, (adjusted *p*-value 0.04956). The QDR2 neighbourhood also contains KGD1, which is associated with LEU3 and annotated to the *lysine degradation* pathway (KEGG 2018), and FKH1, which is annotated to *DNA-binding transcription activator activity, RNA polymerase II-specific* together with CAD1 and LEU3, suggesting that SYG1 and YIL054W might take part in membrane transport processes involving QDR2, that are orchestrated by LEU3 and CAD1 according to cellular amino acids and cations availability.

Another interesting example is gene TPI1 (Figure 8C), a glycolytic enzyme which contains in its cosM multiplex neighbourhood the uncharacterised proteins YDR514C and YMR102C and the transport gene ATP19, subunit of the the mitochondrial F1F0 ATP synthase. The mRNA half-life of TPI1 is regulated by iron availability which is decreased in the ion knock-out profiles of the strains while molybdenum is variably decumulated. This behaviour is characteristic of mRNA sequestrated by P-bodies (processing bodies), which are sites where, under stress conditions, nontranslating mRNA is degraded or stored to return back to translated when the cell enters stress recovery [60]. TPI1 has been experimentally observed to localise in P-bodies under glucose depletion in *S. cerevisiae* via chemical cross-linking coupled to affinity purification (cCLAP) [61], together with other subunits of the F1F0 ATP synthase complex (ATP11, ATP14, ATP20). YDR514C and YMR102C have been observed to physically interact in *S. cerevisiae* with Ccr4, the core subunit of the Ccr4-Not complex, which is involved in the regulation of translation and decay of specific mRNAs and is the main cytoplasmic deadenylase in *S cerevisiae*, and with its associated protein Dhh1 [62]. Ccr4 and Dhh1 associate with mRNAs whose abundance increases during nutrient starvation, and those that fluctuate during metabolic and oxygen consumption cycles. Moreover, there is experimental evidence that YMR102C mRNA is sequestrated by P-bodies under glucose stress, Ca ${}^{2+}$ stress, and Na ${}^{+}$ stress [61]. Given that YDR514C, ATP19, and TPI localise to mitochondria, and that inhibition of TPI1 is known to stimulate the pentose phosphate pathway and to increase antioxidative metabolism [63], the scenario described suggests the idea that YDR514C and YMR102C, together with TPI1, might be activated in the cell to reshape and switch the metabolism in response to ions/nutrients related stress that might cause mitochondrial disruption and aerobic inefficiency. This hypothesis is supported by the ion overexpression profiles of these genes, in which the concentration level of potential toxic species like Cd and As is very low, as well as by experimental evidence that YMR102C is involved in galactose metabolism in the haploid *S cerevisiae* Cd-resistant strain (EC9-8, that tolerates high levels of cadmium) [64], and significantly overexpressed in the ethanol-tolerant strain Y-50316 [65].

As a last example of hypothesis formulation, we considered the cosH multiplex of the Golgi transport gene GCS1 (Figure 8D, left). We found that HRT3, YLR352W, and ROY1 physically interact with Skp1 to form SCF-ubiquitin ligase complexes similar to F-box proteins, despite the fact that they lack an identifiable F-box domain [66]. Gcs1 contains a ArfGAP1 lipid packing sensor (ALPS) motif that binds to lipid membranes to recruit coat complexes whose role is to generate carrier vesicles that mediate transport of proteins and lipids between intracellular compartments. This ALPS motif couples the activity of GCS1 with the curvature of lipid membranes and allows GCS1 to control the assembly and the dynamics of the COPI coat complex, analogously to its human homologue Arf1GAP1 [67]. Interestingly, NUP133, which is found in the neighbourhood of GCS1, is also a membrane curvature sensors and it encodes the same ALPS motif in the Nup84p subcomplex of the nuclear pore complex (NCP) [68]. Moreover, it has been shown that the F-box protein Rcy1 is required for recycling of Snc1 to the Golgi, although its precise mechanism is unknown [69]. An intriguing hypothesis suggested by the ionome–metabolome multilayer is that HRT3, ROY1, and YLR352W might take part in machinery controlling recycling of plasma membrane proteins similarly to RCY1. The ability of the Rcy1-Skp1 complex to recycle independently of the cullin subunit makes this hypothesis plausible even in the absence of recognised E3 activity by Hrt3, Ylr352w, and Roy1. These pseudo-F-box-Skp1 complexes could be specifically activated through different stress factors. Indeed, other genes in the subnetwork include general regulators of ion homeostasis (boron efflux transporter BOR1 and the potassium transport system TRK1). NVJ1, which promotes the formation of ER–vacuole junctions that can expand in response to starvation and regulate the production of lipid droplets [70]. TGL3, whose expression is known to be reduced in the absence of lipid droplets. PDR3, a gene of the conserved pleiotropic drug resistance (PDR) pathway, that, together with PDR1, regulates more than half of the known pumps transporting potentially harmful chemicals outside of the cell membrane, is known to be overexpressed in response to the loss of CCW12 (also present in the subnetwork) which is crucial for wall integrity [71]. GLO1 is also of interest because is known to be regulated by osmotic stress and to process glutathione, a sulfur compound that is synthesised in yeast as a cadmium detoxifying agent and whose synthesis has been recently shown to be mediated by an SCF ligase complex (SCF-Met30) [72].

As a general remark, besides the proposed hypotheses, the clusters of genes discussed, which are not present in the corresponding PCC multilayer network (not shown), confirmed that these measures have the potential to reveal aspects of the complex interplay between metabolism, ion homeostasis, and molecular transport, and can be used as novel analytical tools to quantify genetic similarity on the base of the altered levels of nutrients, such as amino acids and ions, that can be used as footprints of the global impact of altered genetic functions (loss or overexpression) in the cell.

## 4. Discussion

Network science has played a fundamental role in the development of the field of systems biology by providing analytical tools to reveal and characterise protein and genetic interaction maps and the relations between metabolic pathways and functional landscapes of many biological systems [73,74,75]. However, with the advent of the multi-omics era there is recognition that single isolated biological networks are insufficient to describe functional genetic patterns that arise from the multiple levels of complexity of the cell (genome, epigenome, transcriptome, metabolome, proteome, lipidome, ionome) [76]. Network analysis can provide advanced and powerful mathematical frameworks, such as multi-layer networks, to integrate multiple omics data efficiently and in the most intuitive way. In this article, we have focused on the problem of inferring and integrating association networks between genes from omic data sets containing a relatively small number (order O(10)) of biological signatures, profiled for almost all single non-essential gene mutants. These signatures contain comprehensive information on the intracellular concentration of elements or of classes of metabolites, and they present patterns of correlations that reflect those biological and biochemical processes inside the cell in which these concentrations play a fundamental role. The importance of these omic data lies in the fact that the associated studies and methodologies have been proposed as functional omic approaches alternative to the classic functional genomics that can reveal undiscovered relations between genes encoded in the specific omic-related signatures. Extracting informative genetic association networks from these types of short-profile omic data sets is the starting point to apply the tools and algorithms of modern graph theory for revealing new potential functional relations between genes, and from a mathematical perspective this translates into the fundamental issue of assessing reliable similarity scores between the signature vectors. Here, we proposed two pairwise-similarity measures, namely the Mahalanobis cosine, that, to the extent of our knowledge, has never been used before in computational biology, and what we defined as the hybrid Mahalanobis cosine. These two measures can be seen as extensions of the cosine similarity that enforce in different ways additional information from the empirical covariance matrix estimated from the entire set of data under study and can therefore be regarded as providing omic intrinsic or omic adjusted correlations when used to analyse data sets that are at the omic scale. We tested these measures in two fundamental tasks: (1) the inference of genetic relevance networks that can encode in their topology already known biological relationships, and (2) network-based multi-omic integration of short-profile omic data sets, for which multiple evidence of connection across the layers of a multiplex network can indicate potential undiscovered genetic associations. To do that, we developed a methodology that combines extraction and integration of relevance genetic networks based on the robust rank statistic of the similarity scores with cross-referencing from large curated databases of metabolic pathways, genetic interactions, protein–protein interactions, protein complexes co-occurrence, and GO Ontology annotations. We evaluated and compared the performance of the proposed measures against the widely used Pearson correlation coefficient and the standard cosine similarity using three experimental data sets of the ionome and metabolome of the model organism *S. cerevisiae*. Our construction and analysis of the first ionome–metabolome multiplex genetic network of the yeast *S. cerevisiae* indicates that the proposed covariance-based similarity measures, when utilised in the tasks of genetic network inference and network-based omics integration, have the potential to capture alternative and/or additional levels of relations between biological pathways and processes, and they can help to elucidate the function of uncharacterised genes through the inferred multilayer network topology.

The pipeline developed to pre-process the data deliberately removed all samples with any outlier or missing value in their concentration profile. Future work will be directed at investigating the robustness of the two measures proposed (Mahalanobis cosine and hybrid Mahalanobis cosine) to the presence of outliers and to missing value imputation. These questions would require, within the short-profile omic data framework, a combined theoretical and computational analysis of the perturbation spectrum of the eigenvalues of the covariance matrix of multivariate distributions with long tails and correlated dimensions.

As a final remark, the similarity measures proposed define corresponding distance measures through the simple transformation d=(1+s)/2, therefore they can be straightforwardly used in machine learning applications—for example, implemented as alternative metrics into state-of-the-art dimensionality reduction algorithms, such as t-SNE [77] and UMAP [78], and clustering algorithms, including density-based tools [79,80].

## Figures and Tables

**Table 1:** Characteristics of the benchmark data sets considered in the study (after processing raw data). AAFS: average absolute features’ skewness. M/N: number-of-features over the number-of-genes ratio.

	AAFS	M/N
Ionome (ko)	2	0.003
Ionome (oe)	1.45	0.003
Metabolome (aa)	1.15	0.004

**Table 2:** Best performing similarity measures and associated gain in performance in the task of recalling associations from protein–protein interactions (PPI), co-occurrence in protein complexes, co-occurrence in metabolic pathways (KEGG), genetic interactions (BIOGRID), and associations based on GO Ontology Terms (YeastNet GS).The gain in performance is defined as the difference in percentage between the best and the second best true positive rate obtained with the measures under study on a data set.

			Ionome (ko)			
	Top 1000 Scores		Top 10,000 Scores		Top 100,000 Scores	
	Best Performance	Gain	Best Performance	Gain	Best Performance	Gain
PPI	cosH	18.1%	PCC	7.7%	PCC	2.9%
Protein Complexes	cosM	100%	cosM	12.5%	cosH	2.1%
KEGG Pathways	cosH	140.8%	cosH	39.2%	cosH	1%
BIOGRID	cosM	50%	cosM	3%	cosM	5.9%
YeastNet GS	cos	100%	cosH	21.2%	PCC	3.7%
			**Ionome (oe)**			
	**Top 1000 Scores**		**Top 10,000 Scores**		**Top 100,000 Scores**	
	**Best Performance**	**Gain**	**Best Performance**	**Gain**	**Best Performance**	**Gain**
PPI	cosM	60%	PCC	16%	PCC	2.9%
Protein Complexes	cosH	inf	cosM	50%	cosH	2.1%
KEGG Pathways	cosM	7.7%	cosM	3.5%	cosH	1%
BIOGRID	cosH	7.1%	cosM	2.5%	cosM	5.9%
YeastNet GS	cos	14.3%	PCC	2%	PCC	3.7%
			**Metabolome (aa)**			
	**Top 1000 Scores**		**Top 10,000 Scores**		**Top 100,000 Scores**	
	**Best Performance**	**Gain**	**Best Performance**	**Gain**	**Best Performance**	**Gain**
PPI	cosM	146.7%	cosM	91.1%	cosM	41.8%
Protein Complexes	cosM	200%	cosM	80%	cosM	35.1%
KEGG Pathways	cosM	44%	cosM	8.6%	cosM	4.8%
BIOGRID	PCC	0%	cos	0.7%	cos	1.7%
YeastNet GS	cosH	16.7%	cos	8%	cosH	1.8%

**Figure A5:**
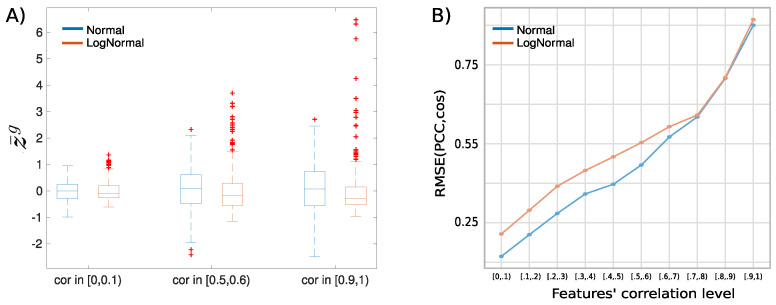
Analysis of the effect of the features correlation and skewness on the discrepancy between the PCC profile similarity and the cos profile similarity. (Panel **A**) boxplots of the average profile values ${\bar{z}}^{g}$ measured on synthetic data sets of n=300 profiles of M=10 Normal features (blue) or log-Normal features (orange) at different levels of features’ correlation. (Panel **B**) from the same synthetic data sets we derived the empirical curve of the root–mean–square error (RMSE) of the scores measured with the PCC with respect to the scores measured with cos in function of the correlation range of the features for both Normal (blue) and log-Normal, skewed features (orange).

**Figure A7:**
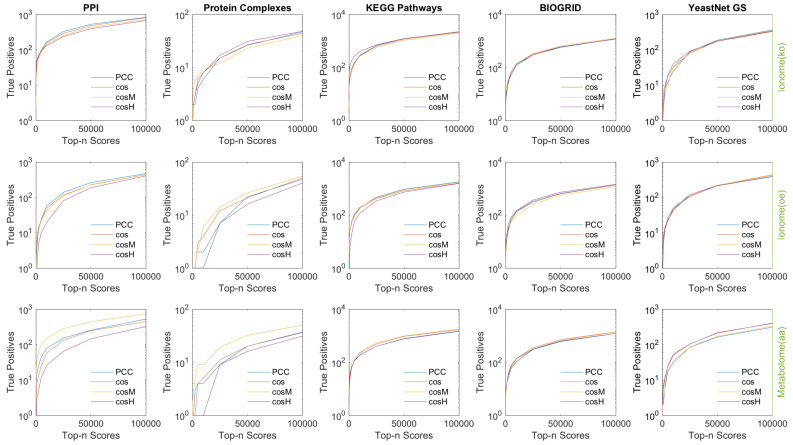
Number of true positive genetic associations from different ground-truth databases (protein–protein Interactions, co-occurrence in protein complexes, co-occurrence in KEGG metabolic pathways, BIOGRID genetic interactions, and associations from YeastNet Gold Standard) retrieved by the similarity measures under study in function of the top *n* most significant scores considered for all three experimental data sets used as benchmarks in this work.

## Data and Codes Availability

No data has been generated during this study. Processed data files and codes can be found at https://github.com/Jaia89/Yeast_Ionome_Metabolome.

## Appendix A. Mahalanobis Vector Space

Let us consider two generic vectors $x,y\in R^{m}$ in a vector space and write the cosine of the angle between them:

(A1) $$cos\left(\theta \right)=\frac{\langle xy\rangle }{{\left||x|\right|}_{2}{\left||y|\right|}_{2}}.$$

This formula implies that the metric of the space is Euclidean; indeed, ${\left||x|\right|}_{2}$ is the $l_{2}$ Euclidean norm. We can write Equation (A1) because a vector space with the Euclidean metric is not just a normed vector space, in which the Euclidean norm satisfies the following three properties:

$$\begin{array}{cc} \left||x|\right| & \geq 0\;\mathrm{and}\;||x||=0\;\mathrm{only if}\;x=0\\ \left||\alpha x|\right| & =|\alpha |||x||\;\mathrm{for any scalar}\;\alpha \\ \left||x+y|\right| & \geq ||x||+||y||\;\mathrm{for any vectors}\;x\mathrm{and}y\;(\mathrm{triangular inequality}), \end{array}$$

but an inner product space, in which also the parallelogram equality

(A2) $$||x+{y||}^{2}-||x-{y||}^{2}={2||x||}^{2}+{2||y||}^{2}$$

is satisfied for any vectors x and y in the space. In fact, the parallelogram equality is a necessary and sufficient condition for the existence of a inner product corresponding to a given norm. When Equation (A2) holds, the normed space is a vector space with an additional structure, the inner product <·>, which is defined by the formula

(A3) $$\langle xy\rangle =\frac{||x+{y||}^{2}-||x-{y||}^{2}}{4},$$

and which naturally induces the associated norm:

(A4) $$\left||x|\right|=\sqrt{\langle xx\rangle }.$$

In the case of the Euclidean metric

$$\begin{array}{cc} \langle xy\rangle & =\frac{||x+{y||}_{2}^{2}-||x-{y||}_{2}^{2}}{4}=\\ \; & =\frac{\sum_{i}{\left(x_{i}+y_{i}\right)}^{2}-{\left(x_{i}-y_{i}\right)}^{2}}{4}=\\ \; & =\frac{\sum_{i=1}^{m}\left(x_{i}^{2}+y_{i}^{2}+2xy-x_{i}^{2}-y_{i}^{2}+2xy\right)}{4}=\sum_{i=1}^{m}x_{i}y_{i}. \end{array}$$

The inner product allows the rigorous introduction of the intuitive geometrical notion of the angle between two vectors, and to define the cosine similarity (Equation (A1)). Now we consider the Mahalanobis metric [28], defined as:

(A5) $$d\left(x,y\right)=\sqrt{\left(x^{T}C^{-1}y\right)}$$

where *C* is a generic covariance matrix. Since any covariance matrix *C* is always semi-positive defined, then, the Mahalanobis metric also satisfies the parallelogram equality, and the formula for the inner product can be rigorously derived:

$$\begin{array}{cc} \frac{||x+{y||}_{M}^{2}-||x-{y||}_{M}^{2}}{4} & =\frac{\sum_{i}\lambda_{i}^{-1}{\left(x_{i}+y_{i}\right)}^{2}-\lambda_{i}^{-1}{\left(x_{i}-y_{i}\right)}^{2}}{4}=\\ \; & =\frac{\sum_{i=1}^{m}\lambda_{i}^{-1}\left(x_{i}^{2}+y_{i}^{2}+2xy-x_{i}^{2}-y_{i}^{2}+2xy\right)}{4}=\\ \; & =\sum_{i=1}^{m}\lambda_{i}^{-1}x_{i}y_{i}\equiv {\langle xy\rangle }_{M}. \end{array}$$

Therefore, the Mahalanobis metric induces an inner product space and an associated cosine measure:

(A6) $$cosM\left(x,y\right)=\frac{{\langle xy\rangle }_{M}}{{\left||x|\right|}_{M}{\left||y|\right|}_{M}}=\frac{\sum_{j=1}^{m}x_{j}^{\prime }y_{j}\lambda_{j}^{-1}}{\sqrt{\sum_{j=1}^{m}{x_{j}^{\prime }}^{2}\lambda_{j}^{-1}}\sqrt{\sum_{j=1}^{m}{y_{j}^{\prime }}^{2}\lambda_{j}^{-1}}}$$

where $x_{j}^{\prime }$ are the elements of the vector x in the base *U* where the covariance matrix is diagonal with eigenvalues $\lambda_{j}$

(A7) $$\Lambda =U^{-1}CU=\left(\begin{array}{ccc} \lambda_{1} & \cdots & 0\\ \vdots & \ddots & \vdots \\ 0 & \cdots & \lambda_{m} \end{array}\right).$$

## Appendix B. Data Processing Pipeline

All raw data sets come in the form of profile vectors w containing concentration values of *M* biological signatures:

(A8) $$w^{g,r}=\left(w_{1}^{g,r},\ldots ,w_{M}^{g,r}\right).$$

where *g* is the mutant index and $r\in (1,\ldots ,R_{g})$ is the replicate index, $R_{g}$ being the number of replicates of mutant *g*. Gene deletion profiles are mostly in replicates of four in the ionome data sets, while in the metabolome data set, only a single amino acid profile is available for most mutants. To pre-process the raw data, we perform the following operations:

*Log-transformation.* A logarithmic transformation is applied to all the profile vectors:

(A9) $${\tilde{w}}^{g,r}=log\left(w^{g,r}\right).$$

*Median batch normalisation.* Each profile element is normalised by the median value of all the concentration values of the corresponding signature *i* measured in the same *p* plate (ionome data) or *p* batch (metabolome data). If $I_{p}$ is the ensamble of mutants measured on plate/batch *p*, then:

(A10) $$x_{i}^{g,r}={\tilde{w}}_{i}^{g(p),r}-\mathrm{median}\left({\left\{{\tilde{w}}_{i}^{g,r}\right\}}_{g,r\in I_{p}}\right)$$

*Outlier detection and removal.* All normalised concentration values $x_{i}^{g,r}$ larger than three or smaller than −3 (corresponding to an absolute log fold-change of 3 with respect to the median value of their plate/batch) are labelled as outliers. These extreme concentration values are unlikely to have any biological explanation and are reasonably considered to result from technical (e.g., mass spectrometer performance) or methodological (e.g., sample preparation) error. All profiles containing at least one outlier value are removed from the data set.

**Table A1:** Statistics of outliers detection and removal for the data sets analysed.

	Outliers	Samples Removed	Mutants Removed	% Samples Removed
Ionome (KO)	211	166	0	0.62
Ionome (OE)	1218	271	1	1.13
Metabolome (AA)	136	48	44	1.03%

*Median gene profile.* To integrate the profile at the mutant level, the median profile over the replicates is extracted for each mutant *g*:

(A11) $$x_{i}^{g}=median\left(\left[x_{i}^{g,1},\ldots ,x_{i}^{g,R_{g}}\right]\right)\;\forall i.$$

*Standardisation.* Each profile element $\bar{i}$ is then standardised by the standard deviation of all concentration values measured across all mutants and replicates:

(A12) $$z_{\bar{i}}^{g}=\frac{x_{\bar{i}}^{g}}{\sigma_{\bar{i}}}$$

were the $\sigma_{\bar{i}}$ are robustly estimated using the mean absolute deviation, through the formula $\mathrm{mad}({\left\{x_{\bar{i}}^{g,r}\right\}}_{g,r})\cdot 1.235$.

*Phenotype profile assessment.* Finally, we do not include in the analysis those genetic profiles that reveal no significant phenotype at the level of any of the features:

(A13) $$\mathrm{discard all mutant profiles}\;g:z_{i}^{g}\leq 0.6\;\forall i.$$

## Appendix C. Synthetic Data Sets

To systematically study the effect of the features’ correlation and that of the features’ skewness on the discrepancy between the profile similarity scores computed with the Pearson’s coefficient and those computed with the cosine similarity we generated synthetic data sets of *n* = 300 profiles of M=10 features. To obtain a desired level of correlation between the features, we adopted the following procedure:

- Step (1): We constructed a square (M×M) correlation matrix *A* with ones on the main diagonal and the [M(M−1)]/2 elements of the upper triangular matrix sampled uniformly at random within a certain correlation interval (e.g., if we want high correlation level, within the interval [0.9,1]). The elements of the lower triangular matrix are imputed from the upper triangular matrix so to have *A* symmetric.
- Step (2): As the eigenvalues of *A* are required to be greater than zero, we computed *S* as the nearest positive definite to the correlation matrix *A*.
- Step (3): We derived the lower triangular *L* matrix of *S* via Cholesky decomposition so to have *S* = LL′.

At this point, it is possible to generate a set of *n* observation of *M* multivariate Normal correlated features with zero means via the matrix product ZL between an n×M matrix *Z* of *M* random N (μ = 0, σ = 1) i.i.d. features, and the M×M matrix *L*. The resulting correlation range of the simulated features will be close enough to those assigned to the matrix *A*. In an analogous way, we also generated synthetic correlated log-Normal features for different levels of feature–feature correlation, for which the empirical distribution of each feature is skewed (by tuning the standard deviation σ of the log-Normal marginal distribution of *Z*, it is possible to have for each feature skewness ≥1.5).

In Figure A4, we report the scatter plots of the profile similarity scores obtained with PCC against those obtained with cos on different synthetic data sets. In the top figures, we show the case of Normal (non-skewed) correlated features for low (range [0,0.1]), intermediate (range [0.5,0.6]), and high (range [0.9,1]) correlation levels, while in the bottom figures, we show the case of log-Normal (skewed) correlated features for the same correlation ranges. In Figure A5B, we plot the trend of the root–mean–square error (RMSE) of the scores measured with the PCC with respect to the scores measured with cos, in function of the correlation range of *A*. As expected, the higher the level of correlation between the features, the more we observe some of the scores returned by the Pearson coefficient and by the cosine similarity to differ. Moreover, the skewness of the features works as an amplifying factor for the RMSE up to relatively high levels (∼0.8) of features’ correlation. In Figure A5A, we show that this discrepancy in the score values is indeed related to the term ${\bar{z}}^{g}$ that appears in the PCC formula. By increasing the level of feature correlation, the variance of the distribution of the average profile values across the *n* observations (centred around ${\bar{z}}^{g}=0$) also increases, so that the fraction of profiles for which $|{\bar{z}}^{g}|>>0$ grows. When the features are extracted from broader, skewed distributions (log-Normal features), the increase in the feature correlation level produces an elongation in the tail of the distribution for positive ${\bar{z}}^{g}$ values that contributes to further increasing the fraction of pairs of profiles $\left(g,g^{\prime }\right)$ for which $PCC\left(z^{g},z^{g^{\prime }}\right)≄cos\left(z^{g},z^{g^{\prime }}\right)$ compared to the case of Normal features.

## Appendix D. False Positive Rate of Genetic Associations

In Figure 5 and Figure A7, we show the true positive rate (TPR) of associations between genes for the measures and the data sets under study with respect to selected ground-truth association sets from databases. A similar analysis to estimate the false positive rate (FPR) would be limiting because the FPR would be relative to each ground truth annotation set. One approach would be to aggregate all the ground truth annotations into a single set; however, the resulting FPR would still implicitly assume that the universe of all biological associations were contained in the aggregated set and it would disregard any possible newly discovered association. Therefore, the FPRs was estimated from the null distributions of the similarity measures by randomly permuting the elements of each column of the N×M matrices of profiles independently. This was repeated 30 times. After each permutation round, the N×N correlation matrix was extracted for each of the measures under study. Once the 30-permutations cycle was completed, all the correlation values related to a specific measure were used to construct a null distribution of the similarity scores for each measure. In order to calculate empirical *p*-values from the null distributions, the similarity threshold discriminating the top *n*-th similarity values for each of the measures was retrieved from the original analysis and the percentages of values in the null distributions that were above those thresholds was computed. The *p*-values (Table A2) indicate the maximum percentage of associations found the networks that are consistent with the null distributions of similarity scores, and, therefore, they represent an upper bound for the false positive rate of associations.

**Table A2:** The maximum false positive rates of genetic associations estimated using empirical *p*-values from the null distributions of the similarity scores. The null distributions are computed through random permutations of the profile matrices for each data set under study.

	PCC	cos	cosM	cosH
**1 K Top Associations**	***p*** **-value (max FPR)**			
Ionome KO	$5.4957\times 10^{-5}$	$5.4026\times 10^{-5}$	$9.2698\times 10^{-5}$	0.000205983
Ionome OE	$0.8823\times 10^{-5}$	$1.0191\times 10^{-5}$	$5.1922\times 10^{-5}$	0
Meatbolome AA	$0.0015\times 10^{-5}$	0	$1.1570\times 10^{-5}$	0
**10 K Top Associations**	***p*** **-value (max FPR)**			
Ionome KO	0.00028	0.000265	0.000729	0.000513
Ionome OE	0.00011	0.000115	0.000557	0.000006
Metabolome AA	$0.00016\times 10^{-8}$	0	0.000408	0
**100 K Top Associations**	***p*** **-value (max FPR)**			
Ionome KO	0.00408	0.00337	0.00756	0.00285
Ionome OE	0.00239	0.00142	0.00555	0.00071
Metabolome AA	0.00012	$0.00075\times 10^{-5}$	0.00795	0
